# Mass-Specific Metabolic Rate and Sperm Competition Determine Sperm Size in Marsupial Mammals

**DOI:** 10.1371/journal.pone.0021244

**Published:** 2011-06-22

**Authors:** Maximiliano Tourmente, Montserrat Gomendio, Eduardo R. S. Roldan

**Affiliations:** Reproductive Ecology and Biology Group, Museo Nacional de Ciencias Naturales (CSIC), Madrid, Spain; University of Manitoba, Canada

## Abstract

Two complementary hypotheses have been proposed to explain variation in sperm size. The first proposes that post-copulatory sexual selection favors an increase in sperm size because it enhances sperm swimming speed, which is an important determinant of fertilization success in competitive contexts. The second hypothesis proposes that mass-specific metabolic rate acts as a constraint, because large animals with low mass-specific metabolic rates will not be able to process resources at the rates needed to produce large sperm. This constraint is expected to be particularly pronounced among mammals, given that this group contains some of the largest species on Earth. We tested these hypotheses among marsupials, a group in which mass-specific metabolic rates are roughly 30% lower than those of eutherian mammals of similar size, leading to the expectation that metabolic rate should be a major constraint. Our findings support both hypotheses because levels of sperm competition are associated with increases in sperm size, but low mass-specific metabolic rate constrains sperm size among large species. We also found that the relationship between sperm size and mass-specific metabolic rate is steeper among marsupials and shallower among eutherian mammals. This finding has two implications: marsupials respond to changes in mass-specific metabolic rate by modifying sperm length to a greater extent, suggesting that they are more constrained by metabolic rate. In addition, for any given mass-specific metabolic rate, marsupials produce longer sperm. We suggest that this is the consequence of marsupials diverting resources away from sperm numbers and into sperm size, due to their efficient sperm transport along the female tract and the existence of mechanisms to protect sperm.

## Introduction

Spermatozoa are cells which show a striking degree of variation in size, ranging from the tiny sperm of the porcupine *Hystrix africaeaustralis* (28 µm) [1] to the gigantic sperm of the fly *Drosophila bifurca* (58,290 µm) [2]. Since the main biological function of sperm is to fertilize, such variation has puzzled researchers for a long time. Two complementary hypotheses have been put forward to explain variation in sperm size.

First, it has been suggested that when females mate with several males, sperm competition would favor the evolution of longer sperm which would swim faster and have greater chances of winning the race to fertilize the ova [3]. Despite the considerable controversy that this hypothesis generated, recent comparative studies have shown that, in several taxa, sperm competition is associated with an increase in sperm dimensions which enhances sperm swimming speed [4]–[8]. Studies on mammals have remained contradictory because although some did find clear associations between sperm size and levels of sperm competition [3], [4] others did not [9], [10]. In addition, studies differed in the component of the sperm cell found to be under the influence of sperm competition, leading to different hypotheses about the relative importance of each component in determining sperm swimming speed. While some studies argued that increased midpiece size would increase mitochondrial volume, resulting in more energy production [7], [11], [12], others argued that increases in whole flagellum length would enhance propelling thrust [3], [4], and others emphasized sperm head elongation that would reduce drag [13]. A recent comparative analysis [8] on a large sample of eutherian mammals has now provided robust evidence that sperm size does increase under sperm competition in this group, and that all sperm components respond by increasing in size because all of them perform complementary roles which are needed to improve swimming speed.

However, a more detailed analysis of different mammalian lineages reveals a more complex picture [14]. Thus, rodents do respond to sperm competition by increasing sperm size (and the size of all sperm components), but other lineages (artiodactyls, carnivores and primates), with larger body sizes, do not. This observation led us to propose a second hypothesis to explain variation in sperm size, named the "metabolic rate constraint hypothesis", which states that small mammals (such as rodents) with high mass-specific metabolic rates have spermatogenic cells capable of processing resources fast enough to respond to sperm competition by producing long sperm, while large mammals are constrained by their low mass-specific metabolic rates and cannot afford the increased costs in terms of energy and time of producing longer sperm [14]. These differences in mass-specific metabolic rates arise because basal metabolic rate scales negatively with body size, so that small mammals have high mass-specific metabolic rates, are more efficient at processing resources, have shorter inter-birth intervals, larger litter sizes and follow a "live fast and die young" strategy, while large mammals have the opposite patterns [15]. In addition, body size is also associated with cellular metabolic rate, so that small mammals have more efficient cells (that are able to process more resources per time unit) than large mammals [16]. This is because properties of cells cannot remain invariant as body size increases, given the associated scaling of whole-organism metabolic rate. Thus, either cellular metabolic rate or cell size must vary with body size, and different cell types follow one strategy or the other depending on their structure and function. Among fast-dividing cells, such as spermatogenic cells, cellular metabolic rate is body-size dependent. As a result, among small mammals spermatogenic cells are likely to have higher metabolic rates which allow them to process energy and resources at a faster rate. When sexual selection intensifies small mammals have spermatogenic cells able to turn resources into sperm rapidly enough to increase sperm size. In contrast, among large mammals, metabolic rate acts as a constraint so spermatogenic cells are unable to respond to sexual selection by producing longer sperm. We tested this hypothesis among eutherian mammals, and found that species with large body sizes and low mass-specific metabolic rate have uniformly small sperm. In contrast, small-bodied species have a large range of sperm sizes because when there is no sperm competition sperm remain small, but as sperm competition intensifies species respond by increasing sperm size [14]. Such increases result in sperm sizes four times larger than those found among large mammals. These findings seem to be driven by differences between rodents and large body-sized mammals, but further analyses are needed to test the generality of this hypothesis.

In this study we test both hypotheses in marsupials (Infraclass: Metatheria), which constitute a monophyletic group considered to be the sister taxon of eutherian mammals. This group seems to be a good model because species differ in levels of sperm competition to a considerable degree (see reviews in [17]–[20]). In addition, comparative studies have revealed that marsupials show extreme variation in sperm length [18], including the mammalian species with the longest sperm (*Tarsipes rostratus*: 350 µm). Marsupials differ from eutherian mammals in that their basal metabolic rate is about 30% lower for any given body size [21], [22] and is more closely associated to body size [22], suggesting that any constraint related to metabolic rate-body size scaling would have a major impact on marsupial physiology. The fact that studies on marsupial sperm size have found a negative effect of body size upon the length of the sperm [18] supports this idea.

## Results

### Marsupial sperm

The dataset analyzed in this study is presented in Table S1, and a summary of information on body mass, relative testes mass and sperm dimensions is shown in Table S2.

Among the 28 marsupial species analyzed, total sperm length ranged from 79.50 µm to 349.44 µm (CV = 45.04) with a mean value of 162.67 µm. Sperm head length represented a mean 4.9% of the total sperm length, ranging from 4.10 µm to 12.80 µm (CV = 34.20). Midpiece accounted for a mean 11.45% of total sperm length and showed the highest variability of all sperm components (CV = 102.26), ranging from 6.90 µm to 88.50 µm. The principal piece comprised a mean 76.63% of total sperm length (range: 50.00–248.65 µm; CV = 43.51).

Testes mass was strongly associated to body mass (*R^2^_adj_* = 0.85, p<0.0001) and could be predicted by the following potential equation: testes mass  = 0.033 * body mass^0.64^. Body mass (CV = 155.54) and testes mass (CV = 134.47) were highly variable when compared to sperm dimensions. However, relative testes size (mean = 1.21) displayed a similar degree of variability (CV = 56.67) than those of sperm dimensions.

### Effects of sperm competition upon marsupial sperm size

**Figure 1:**
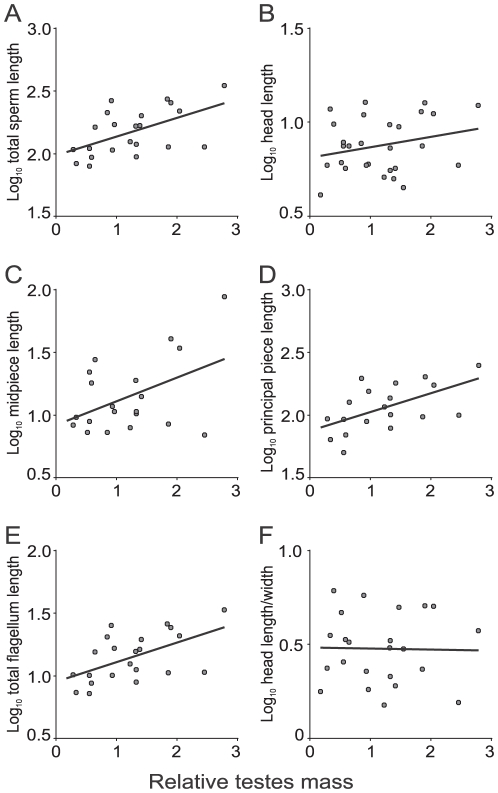
Relations between relative testes mass and sperm dimensions (µm) in marsupial mammals. (A) Total sperm length (*R^2^_adj_* = 0.68, *p* = 0.0071), (B) sperm head length (*R^2^_adj_* = 0.17, *p* = 0.3923), (C) sperm midpiece length (*R^2^_adj_* = 0.24, *p* = 0.1641), (D) sperm principal piece length (*R^2^_adj_* = 0.58, *p* = 0.0253), (E) total sperm flagellum length (*R^2^_adj_* = 0.68, *p* = 0.0059), and (F) sperm head length/width ratio (*R^2^_adj_* = 0.02, *p* = 0.5704).

The phylogenetically controlled GLS multiple regression analysis showed a significant positive association between testes mass corrected for body mass (i.e. relative testes mass) and total sperm length (Table 1; Fig. 1A). When different sperm components were analyzed separately, the same, positive pattern emerged for principal piece length (Table 1; Fig 1D) and total flagellum length (Table 1; Fig 1E). On the other hand, neither head length nor midpiece length were significantly associated with relative testes size (Table 1; Fig 1B and C respectively). There was no relationship between sperm head length/width ratio and relative testes size (Table 1; Fig. 1F).

**Table 1:** Relations between sperm competition and sperm dimensions across marsupials.

Dependent variable	Predictor	Slope	*F*	*p*	*λ*	*r*	CLs	n
Total sperm length	body mass	−0.27	37.57	**<0.0001**	0.462 ^n.s., *^	0.81	**0.69 to 1.59**	22
	testes mass	0.24	9.11	**0.0071**		0.57	**0.20 to 1.10**	
Head length	body mass	−0.11	6.66	**0.0161**	0.997 ^*, n.s.^	0.46	**0.10 to 0.89**	28
	testes mass	0.06	0.76	0.3923		0.17	−0.22 to 0.57	
Midpiece length	body mass	−0.33	6.03	**0.0251**	0.939 ^n.s., n.s.^	0.51	**0.09 to 1.04**	20
	testes mass	0.29	2.12	0.1641		0.33	−0.13 to 0.82	
Principal piece length	body mass	−0.25	20.38	**0.0004**	0.828 ^*, n.s.^	0.75	**0.48 to 1.46**	19
	testes mass	0.21	6.09	**0.0253**		0.53	**0.09 to 1.07**	
Total flagellum length	body mass	−0.28	36.62	**<0.0001**	0.466 ^n.s., *^	0.81	**0.68 to 1.58**	22
	testes mass	0.26	9.62	**0.0059**		0.58	**0.21 to 1.11**	
Head length/head width	body mass	−0.02	2.21	0.1528	0.981 ^*, n.s.^	0.32	−0.11 to 0.76	23
	testes mass	−0.05	0.33	0.5704		0.13	−0.31 to 0.57	

Phylogenetically controlled multiple regression analyses revealing the effect of relative testes mass on sperm dimensions. All variables were log_10_ transformed prior to analysis. The superscripts following the *λ* value indicate significance levels (n.s. *p*>0.05; *p<0.05) in likelihood ratio tests against models with *λ* = 0 (first position) and *λ* = 1 (second position). The effect size *r* calculated from the *F* values and its non-central 95% confidence limits (CLs) are presented. Confidence intervals excluding 0 indicate statistically significant relationships. The *p-*values and CL that indicate statistical significance are shown in bold. Abbreviations: n: number of species in each analysis.

### Effect of mass-specific metabolic rate on sperm size

A significant negative effect of body size on the length of the sperm and all of its components was found regardless of the association of this trait with relative testes size (Table 1). It is important to note that no species with body mass higher than 9 kg produces sperm longer than the mean for this group.

**Figure 2:**
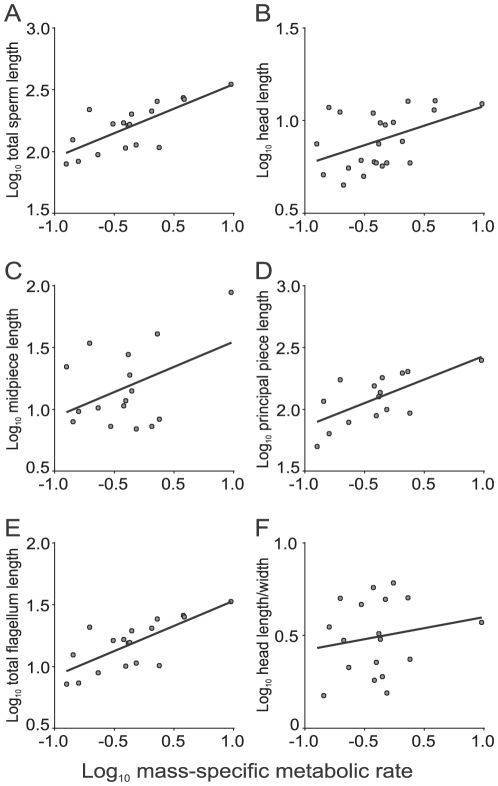
Relations between mass-specific metabolic rate (ml O_2_.h^−1^.g^−1^) and sperm dimensions (µm) in marsupial mammals. (A) Total sperm length (*R^2^_adj_* = 0.64, *p*<0.0001), (B) sperm head length (*R^2^_adj_* = 0.31, *p* = 0.0029), (C) sperm midpiece length (*R^2^_adj_* = 0.28, *p* = 0.0161), (D) sperm principal piece length (*R^2^_adj_* = 0.53, *p* = 0.0013), (E) total sperm flagellum length (*R^2^_adj_* = 0.62, *p* = 0.0001), and (F) sperm head length/width ratio (*R^2^_adj_* = 0.08, *p* = 0.1338).

We found a significant positive relation between mass-specific metabolic rate and total sperm length (Table 2; Fig. 2A) and the length of all sperm components (Table 2; Fig. 2B–E) after correcting for phylogenetic effects. However, head length/width ratio showed no association with mass-specific metabolic rate (Table 2; Fig. 2F).

**Table 2:** Relations between mass-specific metabolic rate (MMR) and sperm dimensions across marsupials.

Dependent variable	Predictor	Slope	*F*	*p*	*λ*	*r*	CLs	n
Total sperm length	MMR	0.39	31.07	**<0.0001**	0.696 ^*, n.s.^	0.81	**0.63 to 1.64**	18
Head length	MMR	0.24	11.25	**0.0029**	0.999 ^*, n.s.^	0.59	**0.24 to 1.12**	23
Midpiece length	MMR	0.57	7.34	**0.0161**	0.969 ^n.s., n.s.^	0.59	**0.13 to 1.22**	16
Principal piece length	MMR	0.38	16.61	**0.0013**	0.930 ^*, n.s.^	0.75	**0.40 to 1.54**	15
Total flagellum length	MMR	0.40	28.87	**0.0001**	0.707 ^*, n.s.^	0.80	**0.60 to 1.61**	18
Head length/head width	MMR	0.20	2.48	0.1338	0.999 ^*, n.s.^	0.37	−0.12 to 0.89	18

Phylogenetically controlled multiple regression analyses revealing the effect of mass-specific metabolic rate on sperm dimensions. All variables were log_10_ transformed prior to analysis. The superscripts following the *λ* value indicate significance levels (n.s. *p*>0.05; *p<0.05) in likelihood ratio tests against models with *λ* = 0 (first position) and *λ* = 1 (second position). The effect size *r* calculated from the *F* values and its non-central 95% confidence limits (CLs) are presented. Confidence intervals excluding 0 indicate statistically significant relationships. The *p-*values and CL that indicate statistical significance are shown in bold. Abbreviations: n: number of species in each analysis; MMR: mass-specific metabolic rate.

**Figure 3:**
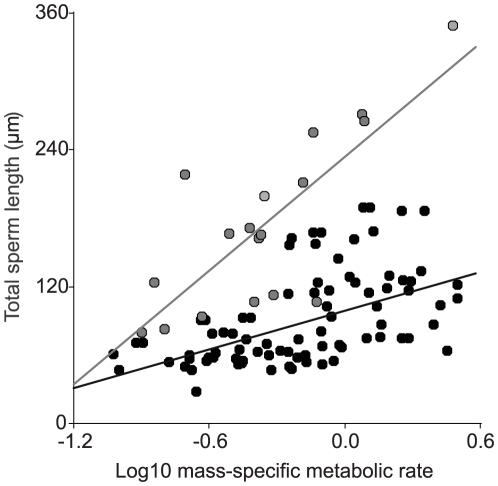
Mass-specific metabolic rate (MSMR – ml O_2_.h^−1^.g^−1^) and total sperm length (TSL –µm) scaling between marsupial and eutherian mammals. Marsupials (grey points and line): TSL = 166.6*MSMR+234.2 (*R^2^_adj_* = 0.59, *p* = 0.0001). Eutherians (black points and line): TSL = 56.0*MSMR+99.2 (*R^2^_adj_* = 0.28, *p*<0.0001). Data for eutherian mammals were from [8].

When we compared marsupials with eutherian mammals we found that the relationship between mass-specific metabolic rate and sperm length is much steeper in the former (Fig. 3). This implies that, at any given mass-specific metabolic rate, marsupials produce longer sperm than eutherian mammals, and that marsupials respond to changes in mass-specific metabolic rate with comparatively greater changes in sperm length than do eutherians.

## Discussion

Our results show that marsupials respond to increased levels of sperm competition by increasing total sperm size. This increase results mainly from an elongation of the flagellum, which is in turn accounted for by an elongation of the principal piece, as only these two sperm components were significantly associated to relative testes size (a proxy of sperm competition levels; reviewed in [8]) in phylogenetically controlled analyses. A longer flagellum could increase sperm velocity by providing more propelling thrust, as it has been shown in other groups [3]–[6], [23]. Recent studies have shown sperm velocity to be related to sperm competition [5], [8] and to constitute a main determinant of fertility in both non-competitive [24]–[26] and competitive contexts [27], [28]. In addition, the effect of sperm competition on the principal piece could result in an increase in energy production. The principal piece is, by far, the largest sperm component among the marsupials, comprising more than 75% of the total sperm length. Among mammals, oxidative phosporylation in the mitochondria has been traditionally considered as the source of energy for sperm movement [29]. Nevertheless, there is evidence that eutherian sperm also produce energy via sperm-specific glycolytic enzymes present in the fibrous sheath of the principal piece [29]–[31]. Furthermore, it has been demonstrated that this sperm component functions as a scaffold for localized proteins involved in signaling cascades [32], [33], which are critical for normal flagellar function [29], [32], [34]. Lastly, since there is evidence of extended periods of sperm storage in the female reproductive tract among marsupial species (families Dasyuridae, Didelphidae and Peramelidae; reviewed in [18]), larger sperm could be advantageous if size was associated with increased survival time [35]. However, there is not enough information on energy production, sperm motility duration, or sperm physiology inside the female reproductive tract among marsupials to test these hypotheses.

In contrast to eutherian mammals [8], sperm competition levels are not associated with differences in head length or mid-piece length among marsupials. The lack of relationship with head length may be due to differences in sperm head shape between marsupials and eutherian mammals (because of a distinct asymmetric positioning of the acrosomal granule in marsupials, their nuclear shape and stability, or the site of insertion of the flagellum [21]). The fact that no relationship was found between relative testes size and midpiece is perhaps more surprising given that this component shows the greatest degree of variation in marsupial sperm, and is considered to be an important determinant of energy supply. However, this finding is in agreement with previous studies which have found no relationship (or a negative relationship) between the length of the midpiece and levels of sperm competition [13].

The range of body mass is narrower in marsupials than in terrestrial eutherians, particularly on the high end (the largest marsupial weighing about 1/100th of the largest terrestrial eutherian) [36]. Recent studies suggest that basal metabolic rate and mass-specific metabolic rates are roughly 30% lower in marsupials, and also less variable, when compared with eutherians of similar size [21], [22] (see also dataset in [36]). Our findings show that, in marsupials, sperm size is associated with mass-specific metabolic rate, which is inversely related to body size, thus supporting the "metabolic rate constraint hypothesis" [14]. In addition, mass-specific metabolic rate influences the length of all sperm components, suggesting that metabolic rate constrains an increase in each and every one of them. Thus, large body-sized species are constrained by the low mass-specific metabolic rates in a similar way than their eutherian counterparts [14].

Nevertheless, there are substantial differences regarding the magnitude of the influence of mass-specific metabolic rate on sperm size between marsupial and eutherian mammals. The slope predicted for this relation for eutherians [14] was considerably lower than the one predicted in the present study for marsupials using the same variables. This difference suggests that decreases in mass-specific metabolic rate associated to larger body size would have a higher impact on sperm size in marsupials. Thus, as marsupials have lower mass-specific metabolic rates than eutherian mammals for any given body size, the constraint on sperm size would be stronger among the former. This may explain why sperm competition levels seem to have no effect on sperm components such as the midpiece which may be costly to enlarge given the mitochondria they contain. Remarkably, our results show that the largest marsupial species that produces sperm longer than the mean for this group has a body size (9 kg) 30.8% smaller than the larger eutherian mammal (13 kg) that produces a sperm longer than the eutherian mean. Therefore, the threshold value of mass-specific metabolic rate necessary to produce relatively large sperm would be reached at lower body sizes in marsupials.

Our study shows that the spermatozoa of marsupials are longer than those of eutherian mammals of similar body size throughout all of the marsupial body size range (total sperm length ranges: 79.50–349.44 µm for marsupials [this study], and 28.30–258.33 µm for eutherians [8]), despite having lower relative testes size and lower mass-specific metabolic rate. If we compare the predicted testes size equations for marsupials (testes mass  = 0.033 * body mass^0.64^) and eutherians (testes mass  = 0.060 * body mass^0.63^; source: dataset in [8]), marsupials have a predicted testes size approximately 40% smaller than eutherian mammals of equivalent size. Testes size in relation to body size is directly associated with sperm production [37]–[39], so these differences are likely to explain the lower sperm numbers found in marsupials (reviewed in [18], [19]). Theoretical studies [35], [40], [41] predict that, since sperm is energetically costly [42]–[46], and ejaculate expenditure can be defined as the product between sperm number and sperm size [47], decreasing sperm numbers could result in more resources available for sperm size. Marsupials exhibit extremely efficient rates of transport of ejaculated sperm to the fertilization site (proportion of ejaculated sperm reaching the oviduct up to four orders of magnitude higher than in some eutherian mammals), which is suspected to be a consequence of the unusual sinusoidal sperm movement (reviewed in [18], [19]). In addition, marsupials have evolved some protective measures, such as the ability to store sperm and the formation of sperm pairs in some species which protects the integrity of the acrosome [48]. Thus, resources could be diverted away from producing higher sperm numbers and allocated to greater sperm size.

In conclusion, our results present phylogenetically robust evidence indicating that sperm competition favors an increase in sperm size, while mass-specific metabolic rate acts as a constraint, among marsupials. Compared to eutherian mammals, marsupials have small testes for their body size and lower mass-specific metabolic rates, suggesting that the latter acts as a major constraint. However, marsupials produce longer sperm than eutherian mammals, presumably because efficient transport along the female tract and protective measures allows them to divert resources away from sperm numbers and into sperm size.

## Materials and Methods

### Sperm competition and sperm design

Data on body mass (g), testes mass (g) and sperm dimensions (µm) were obtained from the literature for 28 species (11 families) of marsupials (see Table S1 for data and references). Sperm dimensions included total sperm length (TSL), head length (HL), head width (HW), midpiece length (MPL), principal piece length (PPL), and total flagellum length (TFL). We also calculated the ratio HL/HW. Additionally, data on basal metabolic rate (BMR) (ml O_2_ . h^−1^) were obtained for a subset of 23 species (11 families) (Table S1). Mass-specific metabolic rate (ml O_2_ . h^−1^ . g^−1^) was calculated as the ratio between basal metabolic rate and body mass. Since body mass measurements taken to determine relative testes size belong to mature males (BM) and basal metabolic rates usually represent a mean value for the species, we used additional body mass measurements (BM2 in table S1) from the same sources of BMR values to calculate mass-specific metabolic rate. Finally, data on the mating system of 17 species were obtained (see Table S1 for data and references) and classified in two categories (polyandrous or monandrous) according to the conclusions of the literature used (Table S1).

### Data analyses

To test whether levels of sperm competition were associated with sperm dimensions, we carried out multiple regression analyses with HL, MPL, PPL, TFL, TSL and HL/HW ratio for all species as dependent variable and relative testes mass as predictor. Relative testes mass was used as a proxy for sperm competition since it is correlated with sperm competition levels in mammals [49], [50]. Additionally, preliminary analyses of our dataset revealed that polyandrous species had larger testes than monandrous species (GLS *p* = 0.0365, *F* = 5.43) when controlling for body size effect. To accurately represent relative testes mass as a measure of sperm competition, we performed multiple regression analyses including both testes mass and body mass as predictors of sperm dimensions. Given that predictor variables were non orthogonal, multiple regression analysis was performed using a sequential (Type I) sum of squares, in which the predictor variables were added to the model in the following order: body mass, testes mass.

To test whether sperm size is associated to mass specific metabolic rate, we performed single linear regression analyses using sperm dimensions (TSL, HL, MPL, PPL, TFL, HL/HW) as dependent variables and mass-specific metabolic rate as predictor. All variables were log_10_-transformed prior to analysis.

Since species data may not be free of phylogenetic association and they may share character values as a result of a common ancestry rather than independent evolution [51], [52], we performed our regressions using a generalized least-squares (GLS) approach in a phylogenetic framework [53]. This method estimates a phylogenetic scaling parameter lambda (*λ*), which represents the transformation that makes the data fit the Brownian motion evolutionary model. If *λ* values are close to 0, the variables are likely to have evolved independently of phylogeny, whereas *λ* values close to 1 indicate strong phylogenetic association of the variables. As an advantage, GLS allows a variable degree of phylogenetic correction according to each tested model, accounting for differences in the level of phylogenetic association between different traits. The estimation of *λ* values and GLS analyses were performed using a code written by R. Freckleton for the statistical package R v.2.8.1 (R Foundation for Statistical Computing 2009) and the maximum likelihood value of *λ* was compared against models with *λ* = 1 and *λ* = 0.

Due to the unavailability of a complete phylogeny for all the species analyzed, a phylogenetic reconstruction was used (Fig. S1). Morphological and molecular trees constructed for the Metatheria were used to determine the phylogenetic position of the higher groups (orders and families) [54], [55], and group-specific molecular phylogenies were used in the case of the groups that accounted for more than two species, namely, Dasyuromorpha [56], Peramelemorpha [56], and Diprotodontia [57]–[59].

All statistical analyses were conducted with R v.2.8.1, and *p*-values were considered statistically significant at α<0.05. We avoided an increase in the chances of commiting type II errors [60] by avoiding the use of Bonferroni correction. In its place, we calculated the effect size *r* from *F* values [61]–[63] obtained from the GLS model; effect sizes ≥0.5 were considered large [64]. Finally, we calculated the non-central confidence limits (CLs) for *r*, which indicate statistical significance if 0 is not contained within the interval [65].

To be able to construct figures including relative testes mass as a variable, values were calculated by dividing the actual testes mass by the predicted testes mass, which was obtained using the method proposed by Kenagy & Trombulak [66]. Since the original paper proposed a general equation for all mammals but only included 2 marsupial species, we constructed the following predicted testes mass equation using the same procedures: testes mass  = 0.033 * body mass^0.64^. However, because this measure has been criticized as an inaccurate index of sperm competition levels due to allometric problems [67], we have not used it in any of the statistical analyses.

## Supporting Information

Figure S1 **Phylogenetic reconstruction for the 28 marsupial mammal species utilized in the GLS analysis.** Phylogenetic position of the higher groups (orders and families) was reconstructed first [54], [55] and group-specific phylogenies were used for Dasyuromorpha and Peramelemorpha [56], and for Diprotodontia [57]–[59].
(EPS)

Table S1 **Sperm dimensions, body mass, testes mass, basal metabolic rate, and mating system in 28 species of marsupials.**
(PDF)

Table S2 **Mean values and ranges of sperm dimensions in 28 species of marsupial mammals.**
(PDF)
