# Supplementary material for: Mass-Specific Metabolic Rate and Sperm Competition Determine Sperm Size in Marsupial Mammals
Source: PLoS One. 2011 Jun 22;6(6):e21244. doi: 10.1371/journal.pone.0021244 (PMC3120838; doi:10.1371/journal.pone.0021244)
Supplement: Table S1 — Sperm dimensions, body mass, testes mass, basal metabolic rate, and mating system in 28 species of marsupials. (PDF) [file pone.0021244.s002.pdf]

Table S1. Sperm dimensions, body mass, testes mass, basal metabolic rate, and mating system in 28 species of marsupials.

Species	Family	Order	HL	HW	MPL	PPL	TFL	TSL	BM	BM2	TM	BMR	MS	Ref. SD	Ref. RTS	Ref. BMR	Ref. MS
Antechinus stuartii	Dasyuridae	Dasyuromorpha	11.40	-	-	-	259.70	271.10	40.00	29.01	0.640	34.81	P	1	2	3	4
Dasycercus cristicauda	Dasyuridae	Dasyuromorpha	9.50	1.90	-	-	-	-	74.50	99.54	0.770	46.62	-	5	2	3	-
Dasykaluta rosamondae	Dasyuridae	Dasyuromorpha	9.80	1.60	-	-	-	-	35.30	35.50	0.126	19.53	-	5	2	6	-
Dasyuroides byrnei	Dasyuridae	Dasyuromorpha	12.70	2.50	40.70	201.40	242.10	254.80	123.60	109.47	1.381	79.64	-	5	2	3	-
Dasyurus viverrinus	Dasyuridae	Dasyuromorpha	11.00	1.90	-	-	-	-	1300.00	1101.49	3.000	409.50	-	5	2	3	-
Sarcophilus harrisi	Dasyuridae	Dasyuromorpha	11.10	2.20	34.40	173.30	207.70	218.40	9000.00	8202.25	24.120	1617.00	P	5	2	3	4
Sminthopsis crassicaudata	Dasyuridae	Dasyuromorpha	12.80	-	-	-	252.10	264.90	14.60	15.98	0.166	19.56	P	7,8	2	3	4
Didelphis virginiana	Didelphidae	Didelphiomorpha	6.10	1.30	7.30	-	-	-	3000.00	2442.08	3.000	719.07	-	9	4	3	-
Monodelphis domestica	Didelphidae	Didelphiomorpha	7.70	-	7.30	197.30	204.60	211.90	110.00	93.45	0.570	61.25	-	7	10	3	-
Macropus agilis	Macropodidae	Diprotodontia	7.50	3.20	8.50	97.00	105.50	113.20	11400.00	-	25.640	-	P	5	10	-	4
Macropus giganteus	Macropodidae	Diprotodontia	7.30	2.20	10.70	100.90	111.60	118.90	40720.00	-	42.020	-	P	5	10	-	4
Macropus robustus	Macropodidae	Diprotodontia	4.50	1.50	-	-	-	-	30000.00	25978.92	40.000	5453.50	P	5	4	3	4
Macropus rufus	Macropodidae	Diprotodontia	5.10	3.40	7.90	116.00	123.90	123.90	39825.00	38968.39	38.180	5568.64	P	5	10	3	4
Wallabia bicolor	Macropodidae	Diprotodontia	7.80	3.05	8.90	92.20	101.10	109.50	31500.00	-	14.751	-	M	5	10	-	10
Petaurus breviceps	Petauridae	Diprotodontia	5.90	2.50	8.30	93.00	101.30	107.10	119.00	120.76	0.200	91.08	M	5	10	3	4
Petaurus norfolcensis	Petauridae	Diprotodontia	4.10	2.30	-	-	-	-	180.00	-	0.162	-	-	5	10	-	-
Trichosurus vulpecula	Phalangeridae	Diprotodontia	5.53	2.58	10.26	78.35	88.61	94.17	3350.00	2685.39	8.260	624.33	P	5	10	3	10
Phascolarctos cinereus	Phascolarctidae	Diprotodontia	11.75	3.32	9.60	63.50	73.10	83.01	8150.00	6528.74	3.720	1034.10	M	5	10	3	10
Aepyprymmus rufescens	Potoroidae	Diprotodontia	5.90	2.60	11.70	88.80	100.50	106.40	2400.00	2810.31	4.680	1111.23	-	11	10	3	-
Bettongia penicillata	Potoroidae	Diprotodontia	7.50	2.30	27.80	127.20	155.00	162.50	872.00	1184.35	1.667	492.20	-	11	10	3	-
Potorous tridactylus	Potoroidae	Diprotodontia	9.70	3.20	18.90	137.20	156.10	165.80	1280.00	976.00	4.380	416.40	P	11	10	13	4
Pseudocheirus peregrinus	Pseudocheiridae	Diprotodontia	5.90	3.80	6.90	100.00	106.90	112.80	684.50	895.22	5.425	434.65	M	5	10	3	4
Tarsipes rostratus	Tarsipidae	Diprotodontia	12.29	3.29	88.50	248.65	337.15	349.44	8.90	9.66	0.365	29.00	P	5,12	10	3	10
Lasiorhinus latifrons	Vombatidae	Diprotodontia	7.50	-	22.00	50.00	72.00	79.50	28290.00	26163.80	13.800	3291.20	-	5	10	3	-
Vombatus ursinus	Vombatidae	Diprotodontia	5.70	1.70	18.00	69.00	87.00	93.60	40100.00	-	18.420	-	-	5	10	-	-
Isoodon macrourus	Peramelidae	Peramelemorphia	6.00	3.30	10.70	154.40	165.10	171.10	2300.00	1505.77	4.720	573.87	P	5	10	3	10
Isoodon obesulus	Peramelidae	Peramelemorphia	5.00	-	-	-	162.00	167.00	978.30	717.00	3.880	222.00	P	5	10	13	4
Perameles nasuta	Peramelidae	Peramelemorphia	5.70	3.00	14.00	180.10	194.10	199.80	1000.00	720.26	4.000	318.50	M	5	4	3	4

Abbreviations: HW: sperm head width (μm), HL: sperm head length (μm), MPL: sperm midpiece length (μm), PPL: sperm principal piece length (μm), TFL: total sperm flagellum length (μm), TSL: total sperm length (μm), BM: body mass used for relative testes size calculation (g). BM2: body mass used for mass-specific metabolic rate calculation (g), TM: testes mass (g), BMR: basal metabolic rate (ml O_2/h), SD: sperm dimensions, RTS: relative testes size, MS: mating system, M: monandrous, P: polyandrous.

References

(1) Taggart DA, Temple-Smith PD (1990) An unusual mode of progression in spermatozoa from the Dasyurid marsupial, *Antechinus stuartii*. Reprod Fertil Dev 2: 107-114.

(2) Taggart DA, Shimmin GA, Dickman CR, Breed WG. (2003) Reproductive biology of carnivorous marsupials: Clues to the likelihood of sperm competition. In: Jones M, Dickman CR, Archer M, editors. Predators with pouches: the biology of carnivorous marsupials. Collingwood: CSIRO. pp. 358-375.

(3) Jones KE, Bielby J, Cardillo M, Fritz SA, O'Dell J, Orme CDL, Safi K, Sechrest W, Boakes EH, Carbone C, Connolly C, Cutts MJ, Foster JK, Greyner R, Habib M, Plaster CA, Price SA, Rigby EA, Rist J, Teacher A, Bininda-Emonds ORP, Gittleman JL, Mace GM, Purvis A (2009) PanTheria: a species-level database of life history, ecology, and geography of extant and recently extinct mammals. Ecology 90: 2648.

(4) Rose RW, Nevison CM, Dixson AF (1997) Testes weight, body weight and mating system in marsupials and monotremes. J Zool Lond 243: 523-531.

(5) Cummins JM, Woodall PF (1985) On mammalian sperm dimensions. J Reprod Fert 75: 153-175.

(6) Withers PC, Cooper CE (2009) Thermal, metabolic and hygric physiology of the little red Kaluta, *Dasykaluta rosamondae* (Dasyuromorphia: Dasyuridae). J Mammal 90: 752-760.

(7) Temple-Smith PD (1994) Comparative structure and function of marsupial sperm. In: Marsupial reproduction: gametes, fertilization and early development. Australia: CSIRO.

(8) Breed WG, Leigh CM (1992) Marsupial fertilization: some further ultrastructural observations on the dasyurid *Sminthopsis crassicaudata.* Mol Reprod Dev 32: 277-292.

(9) Temple-Smith PD, Bedford JM (1980) Sperm maturation and the formation of sperm pairs in the epididymis of the opossum *Didelphis virginiana*. J Exp Zool 214: 161-171

(10) Taggart DA, Breed WG, Temple-Smith PD, Purvis A, Shimmin G (1998) Reproduction, mating strategies and sperm competition in marsupials and monotremes. In: Birkhead TR, Møller AP, editors. Sperm competition and sexual selection. San Diego: Academic Press. pp. 623-666.

(11) Taggart DA, Leigh CM, Schultz D, Breed WG (1995) Ultrastructure and motility of spermatozoa in macropodid and potoroidid marsupials. Reprod Fertil Dev 7: 1129-1140.

(12) Woolley PA, Scarlett G (1984) Observations on the reproductive anatomy of male *Tarsipes rostratus* (Marsupialia:Tarsipedidae). In: Smith AP, and Hume ID, editors. Possums and Gliders. Sydney: Australian Mammal Society. pp 445-450.

(13) White CR, Seymour RS (2003) Mammalian basal metabolic rate is proportional to body mass$^{2/3}$. Proc Natl Acad Sci USA 100: 4046-4049.
