# Supplementary material for: Mass-Specific Metabolic Rate and Sperm Competition Determine Sperm Size in Marsupial Mammals
Source: PLoS One. 2011 Jun 22;6(6):e21244. doi: 10.1371/journal.pone.0021244 (PMC3120838; doi:10.1371/journal.pone.0021244)
Supplement: Table S2 — Mean values and ranges of sperm dimensions in 28 species of marsupial mammals. (PDF) [file pone.0021244.s003.pdf]

Table S2. Mean values and ranges of sperm dimensions in 28 species of marsupial mammals.

Dimension	Mean	SD	Minimum	Maximum	CV (%)	%TSL	Increment (%)
Body mass	9173.42	14277.33	8.90	40720.00	155.54	-	457428.09
Testes mass	9.57	12.87	0.13	42.02	134.47	-	32223.08
Relative testes size	1.16	0.67	0.17	2.79	57.66	-	1541.18
Total sperm length	162.67	73.27	79.50	349.44	45.04	-	339.55
Head length	7.96	2.72	4.10	12.80	34.20	4.89	212.20
Midpiece length	18.62	19.02	6.90	88.50	102.16	11.45	1182.61
Principal piece length	124.65	54.23	50.00	248.65	43.51	76.63	397.30
Total flagellum length	154.87	71.41	72.00	337.15	46.11	95.21	368.26

SD: standard deviation, CV: coefficient of variation, %TSL: mean percentage of the total sperm length represented by each sperm component. The mean percentage of increment indicates the difference between the lowest to the highest value among species.
